# *Cercopithifilaria rugosicauda* (Spirurida, Onchocercidae) in a roe deer and ticks from southern Italy^☆^

**DOI:** 10.1016/j.ijppaw.2013.09.009

**Published:** 2013-10-03

**Authors:** Rafael Antonio Nascimento Ramos, Alessio Giannelli, Filipe Dantas-Torres, Egidio Mallia, Giuseppe Passantino, Riccardo Paolo Lia, Maria Stefania Latrofa, Yasen Mutafchiev, Domenico Otranto

**Affiliations:** a Department of Veterinary Medicine, Università degli Studi di Bari, Valenzano, Bari, Italy; b Department of Immunology, Centro de Pesquisa Aggeu Magalhaes, Recife, Brazil; c Parco Regionale Gallipoli Cognato, Piccole Dolomite Lucane, Matera, Italy; d Institute of Biodiversity and Ecosystem Research, Bulgarian Academy of Sciences, Sofia, Bulgaria

**Keywords:** *Cercopithifilaria rugosicauda*, *Ixodes ricinus*, Roe deer, *Capreolus capreolus*

## Abstract

*Cercopithifilaria rugosicauda* (Spirurida, Onchocercidae) is a subcutaneous filarial nematode of the European roe deer (*Capreolus capreolus*) transmitted by *Ixodes ricinus* (Acari, Ixodidae). At the necropsy of a roe deer from the Parco Regionale di Gallipoli Cognato (Basilicata region, southern Italy), two female nematodes of *C. rugosicauda* were found. Following the necropsy, seven skin snips were sampled from different body regions and 96 *I. ricinus* ticks were collected. In addition, 240 ticks were collected by dragging in the enclosure where the roe deer lived. Samples were examined for the presence of *C. rugosicauda* larvae and assayed by PCR targeting cytochrome *c* oxidase subunit 1 (*cox*1, ∼300 bp) and 12S rDNA (∼330 bp) gene fragments. Female nematodes, microfilariae from skin samples and eight third stage larvae (L3) from ticks were morphologically and molecularly identified as *C. rugosicauda*. Phylogenetic analyses clustered this species with other sequences of *Cercopithifilaria* spp. This study represents the first report of *C. rugosicauda* in a roe deer and ticks from Italy and provides new morphological and molecular data on this little known nematode.

## Highlights

• First report of *Cercopithifilaria rugosicauda* in a roe deer and ticks from Italy.
• This study provides new morphological data on this little known nematode.
• The genetic data provide for the first time information on mitochondrial genes of *C. rugosicauda*.

## Graphical abstract

**Figure:**
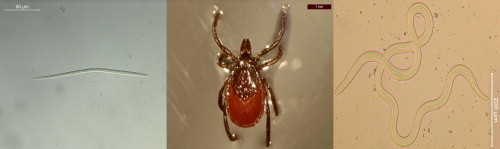

## 1 Introduction

Adult nematodes of the genus *Cercopithifilaria* (Spirurida, Onchocercidae) are parasites of subcutaneous tissues of various mammals (e.g., ruminants, cercopithecid primates, carnivores, rodents and marsupials) in which they release skin dwelling microfilariae (Bain et al., 2002). These parasites are widespread and utilize several species of ixodid ticks as vectors (Bain et al., 2002). Four species of *Cercopithifilaria* have been reported in Europe; *Cercopithifilaria grassii* (Noè, 1907; Otranto et al., 2012a), *Cercopithifilaria bainae* (Otranto et al., 2011, 2013), *Cercopithifilaria* sp. II (Otranto et al., 2012a) in dogs, and *Cercopithifilaria rugosicauda* in roe deer (*Capreolus capreolus*) (Böhm and Supperer, 1953; Winkhardt, 1980). Following the recent retrieval of *C. bainae* in dogs from Italy, Spain and Greece (Otranto et al., 2012b) several studies have been conducted to enhance scientific knowledge on these nematodes (Brianti et al., 2012; Otranto et al., 2012a,c,d). Additionally, until now, *C. rugosicauda* has been reported in a few studies in Austria (Böhm and Supperer, 1953), Hungary (Mészáros and Sugár, 1976), Germany (Winkhardt, 1979, 1980) and France (Bain et al., 1982).

It has been shown that *C. rugosicauda* is vectored by *Ixodes ricinus* (Acari, Ixodidae) and its life cycle completes in approximately 8 weeks in the arthropod vector and 24 weeks in the vertebrate host, in which adults localize in the dermal rump region (Winkhardt, 1980). Although vectors (i.e., *I. ricinus*) and definitive hosts (i.e., *C. capreolus*) of *C. rugosicauda* are prevalent in many geographical areas of Europe, information on the distribution of this filarioid is scant, probably because of the difficulties in detecting microfilariae, and its minor veterinary relevance.

The present study reports, for the first time, *C. rugosicauda* in Italy and provides morphological description of adult and larval stages in roe deer and in tick vectors, respectively. It contributes to the current understanding of the phylogenetic relationship within the genus *Cercopithifilaria*.

## 2 Material and methods

### 2.1 Sample collection

In October 2012, an adult roe deer was found dead in a confined area (100 × 100 m) of the Parco Regionale di Gallipoli Cognato – Piccole Dolomiti Lucane (Basilicata region, southern Italy; 40°32′17″ N, 16°07′20″ E, 844 m above sea level), where it lived with seven other roe deer. This environment was featured by a mixed meadow and forest habitat surrounded by predator-proof fences. The area is located in one of the most important ecological parks in southern Italy, where a rich wildlife fauna (e.g., roe deer, foxes, wolves, wild cats) is observed.

The animal carcass was delivered to the Parasitological Unit of the Department of Veterinary Medicine (University of Bari) and necropsied. Ticks (*n* = 96) were collected from the animals’ fur and put in individual plastic tubes containing 70% ethanol. After skinning, two nematodes were found in subcutaneous tissues. Seven skin snips were sampled from different body regions (i.e., head, inter-scapular, rump, left and right armpits, right and left inguinal regions). Dermal snips were soaked in saline solution for 10 min at 37 °C, and the sediment (20 μl) was searched under microscope for larvae (Otranto et al., 2012d).

Ticks (*n* = 240) were collected also from the environment, in the enclosure where the roe deer lived, by dragging a cotton flannel (90 × 125 cm) for 30 min on the grass, bare soil, and low bushes.

### 2.2 Morphological and molecular identification

Ticks were identified following morphological keys (Manilla, 1998) before being dissected in a drop of 0.9% physiological saline solution and examined under a light microscope (Leica®, DL MB2). The dissected ticks were stored individually in tubes containing phosphate buffered saline (PBS) at −20 °C, until molecular analysis. For light microscopy observations, adult nematodes were cleared and examined in temporary mounts in glycerine. Drawings were made with a light microscope (Leica Microsystems DMLB 2). Adults and microfilariae were photographed and measured using Leica LAS AF version 4.1.

Genomic DNA was extracted from individually dissected ticks (both positive and negative for nematodes) following a protocol previously described (Sangioni et al., 2005), whereas DNA from adult nematode and skin samples was obtained using a commercial kit (DNeasy Blood & Tissue Kit, Qiagen, GmbH, Hilden, Germany). Partial cytochrome *c* oxidase subunit 1 (*cox*1; ∼300 bp) and 12S rDNA (∼330 bp) gene fragments were amplified using primers and reaction conditions as for *C. bainae* (Otranto et al., 2011, 2013). PCR products were purified using Ultrafree-DA columns (Amicon, Millipore; Bedford, USA) and sequenced directly using the Taq DyeDeoxyTerminator Cycle Sequencing Kit (v.2, Applied Biosystems) in an automated sequencer (ABI-PRISM 377). Sequences were aligned using ClustalW program (Larkin et al., 2007) and compared with those available in the GenBank database by Basic Local Alignment Search Tool (BLAST – http://blast.ncbi.nlm.nih.gov/Blast.cgi). The open reading frames (ORFs) of *cox*1 sequences were predicted by conceptual translation into amino acid sequences using the invertebrate mitochondrial code within the MEGA5 software (Tamura et al., 2011). In order to investigate the relationships among filarioids of the Onchocercidae family, sequences of both genes were analyzed with those available in the GenBank database. A phylogeny based on these sequences was constructed using the Maximum Likelihood method (ML) based on the Kimura 2-parameter model (Kimura, 1980), computed by MEGA5 (Tamura et al., 2011). *Thelazia callipaeda* (Spirurida, Thelaziidae) was chosen as an out-group (accession numbers: AJ544882; AJ544858). The nucleotide sequences analysed in this study are available in the GenBank database (12S: KF479369; *cox*1: KF479370).

## 3 Results

**Fig. 1:**
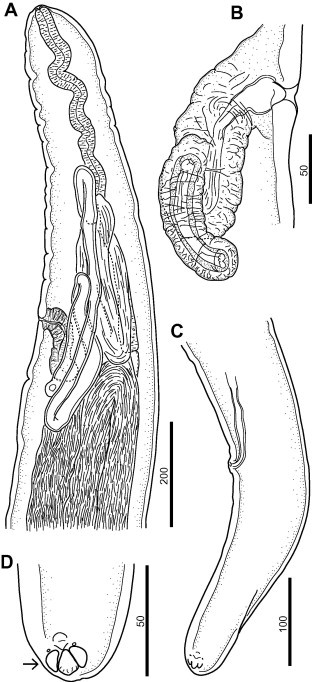
Female *Cercopithifilaria rugosicauda*. (A) Anterior part, lateral view. (B) Vagina, lateral view; note the oesophago-intestinal junction. (C) Tail, lateral view. (D) Tail extremity, ventral view; note ventral protuberances (arrow). Scale-bars in micrometers.

The two female nematodes recovered from subcutaneous tissues of the roe deer (rump and left armpit regions) were identified as *C. rugosicauda*. One specimen was only molecularly processed, because it was damaged, whereas the other was morphologically and molecularly characterised. The female presented the following morphological features: 31 mm long body with a maximum diameter of 220 μm at level of the vulva (Fig. 1A) and anal body diameter of 90 μm; buccal capsule in the form of a flattened ring, leading to oesophagus was 758 μm long and 20 μm wide; vulva situated at 606 μm from anterior extremity; muscular vagina 140 μm long and 60 μm wide (Fig. 1B) followed by a long, convoluted ovejector; tail 250 μm long (Fig. 1C), bearing three short ventrally directed subterminal cuticular lappets (one medioventral and two lateroventral). In addition, two median protuberances are located anterior to the lappets and a pair of phasmids is situated at the base of the lateroventral lappets (Fig. 1D).

**Fig. 2:**
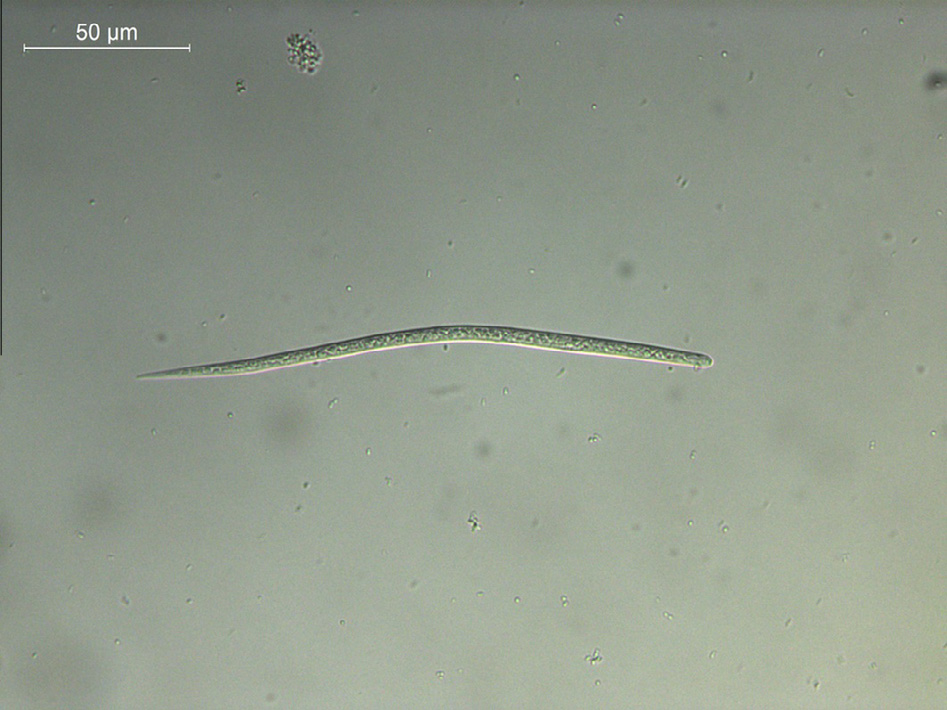
First-stage larvae (L1) of *Cercopithifilaria rugosicauda* retrieved in a skin snip of a roe deer (scale-bar = 50 μm).

Of the seven skin snip sediments observed, two were positive for microfilariae, with six larvae retrieved from the inter-scapular and one from the left armpit region. However, six of the seven skin samples were molecularly positive for microfilariae, but not the one from the right armpit. The dermal microfilariae (*n* = 7) were unsheathed, averaging 178.5 μm (ranging from 173.3 to 185.7 μm) long and 5.6 μm (ranging from 5.0 to 6.1 μm) wide, with rounded anterior end bearing a tiny cephalic hook and pointed tail (Fig. 2).

**Fig. 3:**
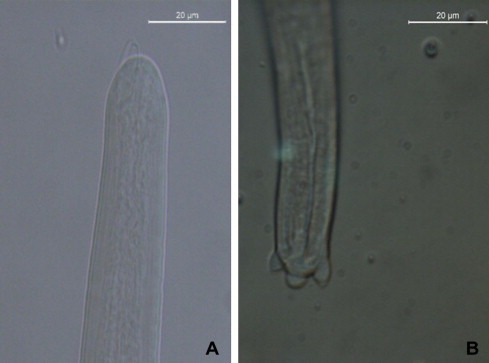
Infective third-stage larvae (L3) of *Cercopithifilaria rugosicauda* found in a dissected nymph of *Ixodes ricinus*. (A) Cephalic region (scale-bar = 20 μm): note the shallow oral cavity lacking buccal capsule. (B) Caudal region (scale-bar = 20 μm): note the presence of three lappets, two conical lateral and one rounded central.

Ticks collected at the necropsy were morphologically identified as *I. ricinus* (*n* = 11 males, *n* = 62 females, *n* = 16 nymphs) and *Haemaphysalis inermis* (*n* = 7 females). Those from the environment were all *I. ricinus* (*n* = 10 males, *n* = 16 females, *n* = 214 nymphs). Six *I. ricinus* ticks collected from the environment were positive under microscopic examination for larvae of *C. rugosicauda* (*n* = 8). One male and three female tested positive using PCR methods, and among the females, one was also positive at microscopic examination. In addition, two nymphs were positive in both microscopic and molecular analyses. All specimens of *I. ricinus* and *H. inermis* collected from the animal were negative for *C. rugusicauda*. At the morphological examination, one L3 was 2.3 mm long and 26 μm wide, with a shallow buccal cavity, a muscular and glandular oesophagus of 141 and 241.9 μm in length, respectively (Fig. 3) and a 88 μm long tail with three terminal rounded caudal lappets (one axial and two smaller lateral).

**Fig. 4:**
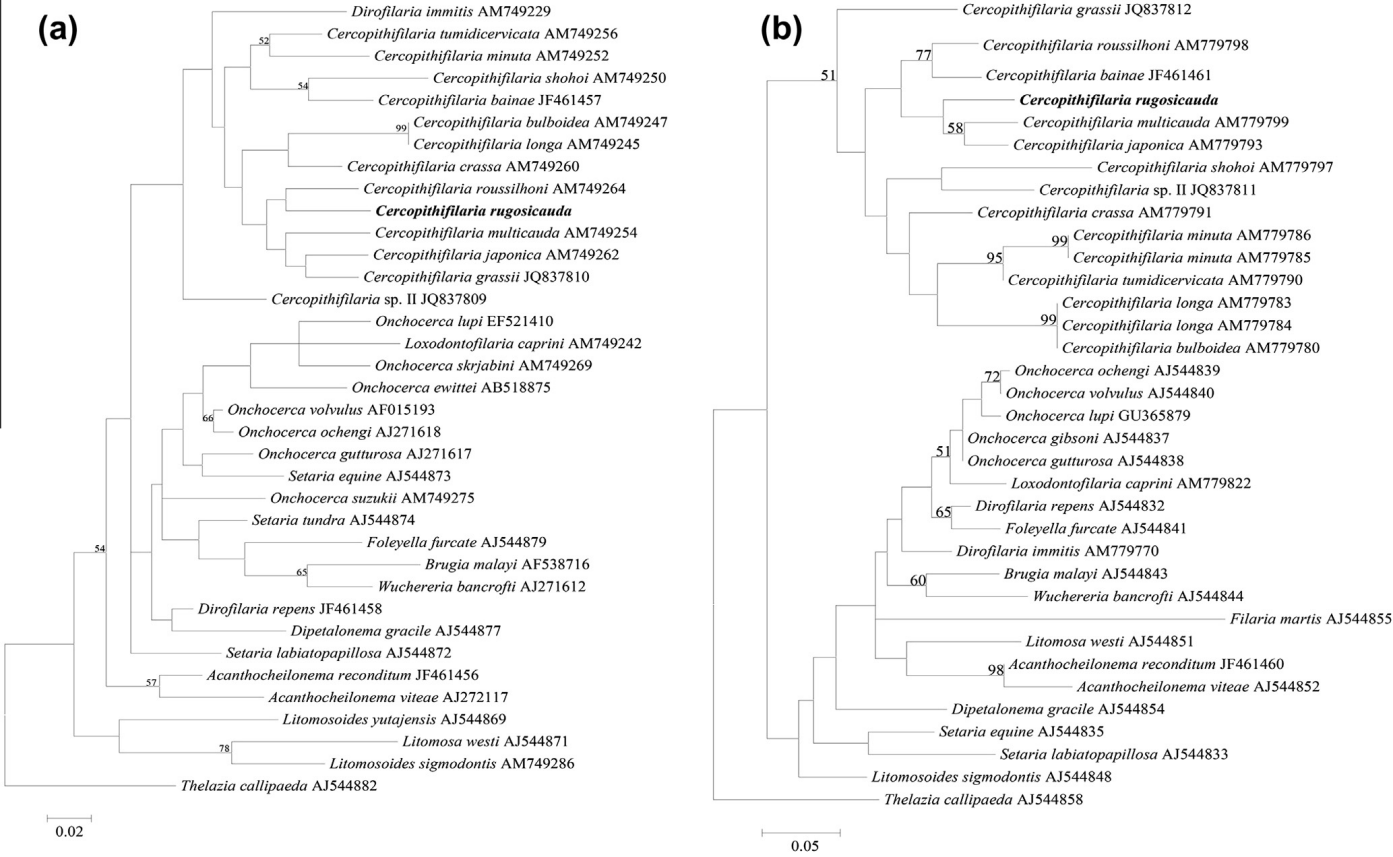
Phylogeny of filarioid Onchocercidae based on *cox*1 (a) and 12S rDNA (b) gene sequences under Maximum Likelihood method, using 8000 replicates bootstrap values. The trees were rooted against *Thelazia callipaeda* (out-group).

Analysis of *cox*1 and 12S rDNA gene sequences revealed 100% homology (i.e., nucleotide conservation) among the adult female, microfilariae and L3 larvae. The highest nucleotide sequence similarity was with *Cercopithifilaria roussilhoni* and *Cercopithifilaria japonica* available in the GenBank database (i.e., *cox*1: 89% and 12S: 88%, respectively). In particular, the molecular analysis, which revealed that *cox*1 sequences aligned over 256 sites, showed a typical AT base composition of 63.7% with a bias at the third codon position of 74.2%, compared with the first and second positions (i.e., 58.1%). The conceptual translation at the third codon position of *cox*1 sequence led to 84 proteins without stop codons. Analogously, the sequences of 12S rDNA genes were aligned over 280 sites with an AT base composition of 76.8%. The phylogenetic analyses of sequences representing both the adult and microfilaria specimens here examined and those available for other onchocercid species were concordant in clustering them with those of other *Cercopithifilaria* spp. available in GenBank as a monophyletic group without the inclusions of other filarial nematodes, for all genes examined (Fig. 4a and b).

## 4 Discussion

This study provides further information on the morphology of *C. rugosicauda*, on its genetic makeup and reports, for the first time, the occurrence of this filarioid in a roe deer and in *I. ricinus* ticks from Italy. Indeed, following the original description of *C. rugosicauda* from roe deer in Austria (Böhm and Supperer, 1953), morphological data of the species has been provided in Hungary (Mészáros and Sugár, 1976) and France (Bain et al., 1982). The morphological characters of the female worm described in this study resemble other *C. rugosicauda* specimens (e.g., the position of the vulva situated anterior to the oesophago-intestinal junction, the markedly increased body diameter posterior to the vulva, the morphology of the vagina, the ovejector convoluted in the region of oesophago-intestinal junction, as well as by the tail bearing three short ventrally directed subterminal lappets). The specimen described here is distinct from those described in Austria by its shorter body (31 *vs* 35–40 mm), smaller distance from the anterior body end to the vulva (600 *vs* 717–761 μm), shorter tail (250 *vs* 380–390 μm) and shorter oesophagus (758 *vs* 850–880 μm). The morphological variations in *C. rugosicauda* specimens from different European localities need additional studies, although they might reflect an intraspecific variability, as demonstrated for other onchocercid species. Indeed, the microfilariae herein described have smaller body dimensions than those described by Böhm and Supperer (1953): 178.5 μm (173.3–185.7 μm) *vs* 212–222 μm long and 5.6 μm (5–6.1 μm) *vs* 6–7 μm wide. However, their dimensions are within the range of variability of those reported by Winkhardt (1980) (165–210 μm long; 5–7 μm wide). Winkhardt (1979, 1980) elucidated the life cycle of *C. rugosicauda* and revealed *I. ricinus* as its proper vector in Germany. The third stage larvae of *C. rugosicauda* described by Winkhardt (1980) are characterized by shorter body length than the larvae measured in the present study (1.4 and 2.0 *vs* 2.3 mm). However, both larvae have a similar morphology. Based on the discussion above, we identified the material studied here as conspecific with *C. rugosicauda*. The geographic range of this species is documented in the temperate climatic zone of Europe, but it is new for the fauna of Italy in an area with a subtropical Mediterranean climate.

The microscopic and molecular examination of seven skin snip samples showed a low concordance with only two samples positive in both tests. A low level of concordance between these analyses was previously suggested in a study about the infection of *C. bainae* in dogs, due to the fact that most microfilariae in the sediment are removed for microscopic examination, reducing the probability of detecting their DNA by PCR (Otranto et al., 2012b). The presence of dermal microfilariae detected during autumn (i.e., October), overlaps with the period in which *I. ricinus* was found in southern Italy (Dantas-Torres and Otranto, 2013a).

The detection of L3 of *C. rugosicauda* in *I. ricinus* nymphs in the present study, along with the results presented by Winkhardt (1980), confirms that this stage acts as a vector of the nematode and that the larval stage of this tick feed on roe deer. These data are of interest as *I. ricinus* immature stages are considered to feed primarily on small and middle-sized mammals, birds and reptiles (Václav et al., 2011; Falchi et al., 2012), whereas adults usually feed on large mammals (e.g., roe deer) (Nuttall and Warburton, 1911; Manilla, 1998; Estrada-Peña et al., 2005). The permanent presence of roe deer in this enclosure during the year might be an important factor influencing local tick abundance and, probably, the short life cycle of these ticks in this area (Dantas-Torres and Otranto, 2013a). The genetic data presented here provide, for the first time, DNA sequences for two mitochondrial genes of *C. rugosicauda*, providing an additional method for identification. Furthermore, the phylogenetic analyses for both mitochondrial markers confirmed the placement of this species within the genus *Cercopithifilaria*, by clustering *C. rugosicauda* in a monophyletic group within other congeners.

As there is no evidence supporting the pathogenic role of *C. rugosicauda*, other studies are needed in order to clarify the impact of this parasite, if any, on the welfare of its wild hosts. Although *I. ricinus* has been established as a vector of *C. rugosicauda*, the role of other ixodid species as vectors cannot be dismissed. Indeed, other tick species (e.g., *Rhipicephalus turanicus*) have been found in the same enclosure from where the roe deer examined came from (Dantas-Torres and Otranto, 2013b). In conclusion, this study represents the southernmost record of *C. rugosicauda* to date. This suggests a wider range for this nematode whose geographical distribution probably overlaps with the distribution of *I. ricinus* and roe deer.
